# Decreasing COVID-19 Risk Factors for Older Adults by Using Digital Technology to Implement a Plant-Based-Diet: An Opinion

**DOI:** 10.2196/25327

**Published:** 2021-07-05

**Authors:** Heidi Lynn Benavides, Christiane Lumachi Meireles, Viola Benavente, Mary Helen Mays, Jing Wang

**Affiliations:** 1 School of Nursing UT Health San Antonio San Antonio, TX United States; 2 Office of Faculty Excellence UT Health San Antonio San Antonio, TX United States

**Keywords:** COVID-19, coronavirus, older adult, plant-based diet, eating patterns, whole foods, Mediterranean diet, obesity, pandemic, ethnic minorities, telehealth, digital technology, racial disparities, aging

## Abstract

A disproportionate number of COVID-19 cases affect older, minority populations. Obese older adults are at higher risk of developing severe COVID-19 complications and lower survival rates, and minority older adults often experience higher rates of obesity. A plant-based diet intervention may improve COVID-19-related modifiable risk factors for obesity. Encouraging the consumption of plant-based diets comprising vegetables, fruits, whole grains, legumes, seeds, and nuts by utilizing community outreach strategies and digital technology can contribute to improving COVID-19 risk factors among this population.

## Introduction

The risk of severe illness with COVID-19 increases with age, with older adults at the highest risk of hospitalization or death [1]. Certain underlying medical conditions increase these risks, including hypertension, heart disease, cancer, type 2 diabetes, chronic kidney disease, and obesity [2]. Minority populations are at higher risk for developing these chronic diseases, thereby increasing their risk of contracting COVID-19 and consequent death. Many factors place minority individuals at risk of contracting the virus, including health care access, socioeconomic status, and frontline worker occupational exposure [3].

The Centers for Disease Control and Prevention reports COVID-19 cases, hospitalizations, and deaths of people of various ethnicities in comparison to White, non-Hispanic persons [3]. American Indian or Alaska Native non-Hispanic persons are high-risk groups for COVID-19, with 1.6 times higher cases, 3.5 times higher hospitalization rates, and 2.4 times higher death rates reported. Black, non-Hispanic individuals are also disproportionately affected by the pandemic, with 1.1 times higher likelihood of contracting COVID-19, 2.8 times higher hospitalization rates, and 1.9 times higher death rates reported. For example, in the District of Columbia, Black individuals constitute 47% of the population, yet they accounted for 74% of COVID-19–related deaths [4]. Hispanic or Latino individuals were 2 times more likely to contract COVID-19, had 3 times higher hospitalization deaths, and were 2.3 times more likely to die [3]. Moreover, Hispanic individuals living in the District of Columbia make up 11% of the population but accounted for 29% of all COVID-19–related deaths [4]. In Texas as well, disparities were noted, as 39% of the population is Hispanic but accounted for a higher COVID-19 death rate of 54%.

The United States is plagued with an obesity epidemic, which has worsened over the recent decades [5]. Obesity and severe obesity are defined as a BMI of 30 kg/m^2^ and ≥40 kg/m^2^, respectively. The rate of obesity in the period from 1999 to 2000 was 30.5%; during 2017-2018, this rate had increased to 42.4%. The trend for severe obesity nearly doubled in 10 years, increasing from 4.7% in 1999-2000 to 9.2% in 2017-2018. Obesity and severe obesity rates have increased, placing a disproportionate burden among communities of color. Non-Hispanic black individuals had the highest prevalence rates of obesity (49.6%), followed by Hispanic individuals (44.8%). By gender, US-born Hispanic or Latino men have the highest obesity rate compared to men of other ethnicities, including those who are foreign-born [6].

Age is also a risk factor known to increase the development of severe illness related to COVID-19, including increasing rates of hospitalization and lowered prognosis of survival [1]. Individuals 65 years and older with a positive COVID-19 laboratory test were noted to have higher hospitalization rates (13.8%) than younger individuals, and those 85 years and older were at even greater risk, with a hospitalization rate of 17.2% [7]. The COVID-19 crisis has highlighted how the cumulative impact of multiple risk factors, such as being older, obese, and a member of a minority group, dramatically increases the probability of hospitalization and death due to COVID-19. For example, older, minority men who smoke and have a BMI ≥35 kg/m^2^ require increased oxygenation while hospitalized [1].

## Support for Plant-Based Diets: Role in Managing Obesity and Related Risk Factors

Reducing obesity and severe obesity is a complex process, including behavior change, diet modifications, and increased in physical activity. Dietary changes that reduce the amount of trans and saturated fat consumed, as well as refined or simple carbohydrates, support healthy weight loss.

Although only 2.4% of the general US population has adopted a plant-based diet, there is a growing interest toward incorporating a more plant-based diet for different reasons, including a healthy lifestyle [8]. Dietary modification is an accessible, measurable, and translatable health behavior. Identifying achievable self-management behaviors that promote and maintain a plant-based diet has been shown to decrease adiposity, BMI, and hemoglobin A_1C_ levels in certain minority groups and older adults [9,10].

A plant-based diet refers to a pattern of food intake that emphasizes consumption of vegetables, fruits, whole grains, legumes, seeds, and nuts, excluding the intake of all animal products or including some animal-based foods such as milk, dairy products, and eggs. Cumulative evidence shows the benefits increasing consumption of plant-based diets has on several chronic diseases such as obesity [11-13]. Healthy plant-based dietary patterns include consumption of foods low in total saturated fat and high in fiber, antioxidants, and phytochemicals [14]. As a result, a number of health and nutrition organizations, as well as evidence-based weight loss programs, recommend foods that are more plant-based. The American Institute for Cancer Research recommends that two-thirds of the total dietary intake include vegetables, fruits, whole grains, and beans [15]. The Academy of Nutrition and Dietetics states that “appropriately planned vegetarian, including vegan diets are healthful, nutritionally adequate, and may provide health benefits for the prevention and treatment of certain diseases” [14]. These diets, the Academy notes, can be appropriate for older adults when balanced to meet recommended daily requirements. A concern with plant-based diets is the lack of vitamin B_12_, which is only available in animal foods. Older adults can have decreased absorption of vitamin B_12_ and may require vitamin B_12_ supplementation, regardless of their overall eating patterns.

Additional support for plant-based diets can be found in the Dietary Guidelines for Americans, 2020-2025, which reports that increasing consumption of vegetables, fruits, and whole grains and adopting vegetarian and Mediterranean eating patterns is healthful for all ages [16]. The Mediterranean Diet and the DASH (Dietary Approach to Stop Hypertension) diet have been linked to healthy dietary patterns focusing on higher consumption of plant-based foods. These dietary plans also recommend a moderate intake of milk and dairy, decreased consumption of red and processed meat, and increased consumption of fish [17,18].

The PREDIMED (Prevención con Dieta Mediterránea) study, comprising a prospective cohort with more than 7000 older participants, associated a preference for plant-derived foods with reduced mortality among older adults with high cardiovascular risk [19]. Although the PREDIMED study shows the benefits of shifting food patterns to a more vegetarian diet in an older population, adherence to the diet, especially in this population, may pose a challenge.

Transition to a plant-based diet may be easier when the foods are consistent with the individual’s culture, religious beliefs, and food preferences. In a study of middle-aged to older South Asian participants in the United States, those who had strong traditional South Asian cultural beliefs maintained their vegetarian diet [20]. In a previous study by Ramal et al [10], Latino patients with type 2 diabetes living in medically underserved areas were educated on the benefits of foods rich in fiber, low in fat, and derived from mostly plant-based sources. Group sessions were used to help participants shift their dietary patterns to a more plant-based one, also helping them to implement and comply with the dietary recommendations. The intervention had a significant impact on the participants’ hemoglobin A_1C_ levels, a reduction in fat intake, and hip circumference. This study showed plant-based foods can be successfully implemented in the Hispanic culture when the individuals and families are aware of the benefits and are supported by their families, community, and health care professionals. Research has shown that Latino or Hispanic participants who followed a more plant-based dietary approach had lower adiposity rates, BMIs, and hemoglobin A_1C_ values with reduced cardiovascular risk [9,10]. Given the growth of and high prevalence of obesity among US Hispanic or Latino populations, especially in older adults with underlying cardiometabolic conditions, a change in dietary patterns has the potential to make a great public health impact and reduce COVID-19 risk and mortality rate [10,21].

The Adventist Health Study 2 sampled members of the Seventh-day Adventist church [22]. A large sampling of non-Hispanic Black adults showed that the absence of obesity improved life expectancy and that a plant-based diet promoted healthy weight, which was associated with increasing longevity. Such positive study findings indicate that this type of nutritional approach is a healthy lifestyle behavior in underserved racial or ethnic subpopulations [23,24].

## Increasing Adoption of Plant-Based Diets

Plant-based nutrition education programs in the United States are scarce. However, there is enough scientific evidence supporting plant-based diets to help combat obesity and associated diseases [11-13], and such programs should be considered for large-scale implementation and effectiveness testing. Community outreach programs and other strategies can help minority older adults adopt plant-based diets to increase consumption of vegetables, fruits, whole grains, and legumes.

Nutrition education is often effective when delivered through the grocery shopping process. Yet, this routine activity may constitute a burden and barrier for older people, especially older adults living in food deserts and poverty-stricken areas. During the COVID-19 pandemic, there has been an increased use of online and curbside pickup for grocery shopping. Nutrition education tips and recommendations can be included in grocery store websites that support increasing plant-based food consumption. This may include highlighting weekly sales items that fit into a plant-based diet as well as simple recipes for preparing these foods, where local cultures and food preferences are considered. Moreover, in situations where the older adult may get assistance from family members with grocery shopping, this information may not only influence the dietary intake of the older adult but may also positively influence the nutritional intake of the family in general.

Community gardens are effective outreach programs implemented in neighborhood community centers and can be tailored to local cultural preferences [25,26]. These programs often increase access to a variety of affordable and healthy foods to older adults, as well as the community at large [27-30]. This concept represents a “farm-to-table” resource for fresh produce, which may be at lower cost or no cost to older adults. Social distancing restrictions during the COVID-19 pandemic have limited opportunities for social interaction at community gardens, as well as contributions to garden development. As more people are immunized against COVID-19, and these opportunities open up, it is likely that participation in community gardens may increase followed by improved vegetable consumption, increased physical activity, and reduced obesity rates [31].

In 2001, the US Department of Agriculture began a Seniors Farmers’ Market Nutrition Program (SFMNP) [32]. The program serves low-income older adults, generally defined as individuals who are at least 60 years old and who have household incomes of not more than 185% of the federal poverty income guidelines with sites located typically at centers and housing locations for older adults. In fiscal year 2017, the SFMNP assisted 811,809 low-income older adults with benefits. SFMNP awards grants to US states, US territories, and Indian Tribal Organizations to provide fresh, nutritious, unprepared, and locally grown fruits, vegetables, herbs, and honey through farmer’s markets, roadside stands, and community-supported agriculture programs [32]. Older adults who qualify for the program should be encouraged to utilize the coupons to help increase their consumption of eligible foods. Transportation may be a barrier for some older adults, thereby decreasing their ability to utilize SFMNP coupons in their community. If available, ride-share programs (eg, Uber and Lyft) may be an option.

The Older American’s Act (OAA) funds 39% of the Meals on Wheels program and is the primary federal legislation supporting social and nutritional needs of vulnerable persons who are 60 years and older [33]. Meals on Wheels America supports over 5000 community nutrition centers for older adults and the delivery of meals to homebound older adults [34]. One myth about the program is that meal choices are not allowed; however, in 2016, Meals on Wheels provided an executive summary that dispelled this myth. The OAA allows food choices and should be promoted among the older adult population. However, each US state develops their own policies and procedures based on the OAA, making interpretations difficult. Older adults should be encouraged to make menu choices to accommodate personal preferences, and plant-based options could be included, especially in forms that are in harmony with the participant’s culture, beliefs, and religion. Encouraging older adults to eat fruits and vegetables through outreach programs such as Meals on Wheels can improve their adoption of a plant-based diet.

## Leveraging Digital Technologies to Promote Plant-Based Diet Intake

The COVID-19 pandemic has significantly increased the use of technology in a broad range of activities such as health care (eg, telemedicine and telehealth); sourcing foods, both ready-to-eat as well as to be prepared (eg, restaurant delivery, curbside grocery pickup); and social and work interactions through platforms such as Zoom and Microsoft Teams. Advances in digital health technologies, telehealth, and internet access can facilitate the delivery of effective plant-based diets and behavioral programs safely to minority, aging populations. These types of modalities decrease the risk of COVID-19 exposure, provide ways to deliver education, and enhance local and community support.

When considering the use of eHealth and technology by older adults, including minority older adults, in general, it is important to realize that many may lack or have low levels of eHealth literacy [35]. Norman and Skinner [36] defined eHealth literacy as “the ability to seek, find, understand, and appraise health information from electronic sources and apply the knowledge gained to addressing or solving a health problem.” eHealth literacy is a combination of six core skills (or literacies): traditional literacy, health literacy, information literacy, scientific literacy, media literacy, and computer literacy. Although proficiency is not needed in all six skill areas, a minimum competency across all skills is considered essential to promote eHealth literacy.

Reducing barriers to technology use among older adults, including those representing minorities, is important to improve uptake and use of available resources. Many older adults face low vision challenges; therefore, understanding how to enlarge text or images on a computer screen can help address this barrier. For those older adults who have less experience in basic computer or smartphone use (eg, how to access the internet, how to access or download an app), having a trusted friend or family member provide a “guided tutorial” on how to complete these steps can help build both knowledge and confidence in the ability to use available technology. Instruction via distance (such as Zoom or Facetime on smartphones) makes this instruction possible even when physically separated. Those older minority adults who are not comfortable with or fluent in English, may find apps and sites that are available in different languages. Although multilanguage websites and applications are more frequently available, some minorities may still find barriers resulting from cultural variations in spelling or word usage. In these cases, a family member, friend, or local public librarian who can navigate these idiomatic challenges may be needed.

Telehealth can enhance digitally delivered education through behavioral coaching with a remote health care provider, dietitian, and/or nurse either individually or by utilizing a shared medical appointment (SMA) approach. SMA is a group patient visit and may last 120 minutes long. An SMA can provide group education by the health care team to help reduce costs and allow more time with the health care team; it has been overwhelmingly supported by the patient [37]. For instance, a group of 10 patients with the same medical diagnoses could have an appointment at the same time with the health care provider, a nurse, and a dietitian, thus allowing more time to be spent with the patient than a typical one-on-one appointment tailoring to each specific condition.

User-friendly, low-literacy apps and online community support platforms are available in various languages to support nutrition behavior modification. These apps typically allow food tracking through capturing pictures and easy access to lifestyle coaches. Such apps can help track calorie-counting, provide information on plant-based meal preparation, and calculate macro and micronutrients. In addition, there are apps to help search for local plant-based diet restaurants, stores, and markets, thus aiding the individual in achieving healthy decision-making. These types of apps are becoming more popular to support the adoption of plant-based diets.

## Conclusions

Promoting plant-based diets by using technology can be beneficial in decreasing modifiable COVID-19 risk factors for minority older adults. Consumption of a mostly plant-based dietary pattern promotes healthier BMI and hemoglobin A_1C_ levels. Community outreach programs such as Meals on Wheels, community gardens, Seniors Farmers’ Market Nutrition Program and other strategies are critically important to help improve obesity and related health considerations. Implementing technology into patient treatment plans by using low-literacy apps and either individual or group virtual health visits could significantly improve health disparities among vulnerable populations and benefit future generations. Emphasizing plant-based diet consumption is promising and needs to be further explored, which may reduce COVID-19–related health disparities and promote healthier weights in minority older adults.

## Abbreviations

DASH: Dietary Approach to Stop Hypertension
OAA: Older American’s Act
PREDIMED: Prevención con Dieta Mediterránea
SMA: shared medical appointment
SFMNP: Seniors Farmers’ Market Nutrition Program
